# Connectomics: A New Direction in Research to Understand the Mechanism of Acupuncture

**DOI:** 10.1155/2014/568429

**Published:** 2014-01-02

**Authors:** Ruirui Sun, Yue Yang, Zhengjie Li, Ying Li, Shirui Cheng, Fang Zeng

**Affiliations:** ^1^The Acupuncture and Tuina School, The 3rd Teaching Hospital, Chengdu University of Traditional Chinese Medicine, No. 37, Shierqiao Road, Chengdu, Sichuan 610075, China; ^2^Psychosomatic Medicine Department, Sichuan Academy of Medical Sciences & Sichuan Provincial People's Hospital, Chengdu, Sichuan 610072, China

## Abstract

Acupuncture has been used to treat various disorders in China and some other eastern countries for thousands of years. Nowadays, acupuncture is gradually accepted as an alternative and complementary method in western countries for its undeniable therapeutic effects. However, its central mechanism is still unclear. It is especially difficult to reveal how different regions in the brain influence one another and how the relationship is among these regions responding to acupuncture treatment. Recently, by applying neuroimaging techniques and network theory, acupuncture studies can make further efforts to investigate the influence of acupuncture on regional cerebral functional connectivity (FC) and the modulation on “acupuncture-related” networks. Connectomics appears to be a new direction in research to further understand the central mechanism underlying acupuncture. In this paper, an overview of connectomics application in acupuncture research will be discussed, with special emphasis on present findings of acupuncture and its influence on cerebral FC. Firstly, the connectomics concept and its significance on acupuncture will be outlined. Secondly, the commonly used brain imaging techniques will be briefly introduced. Thirdly, the influence of acupuncture on FC will be discussed in greater detail. Finally, the possible direction in forthcoming research will be reviewed by analyzing the limitation of present studies.

## 1. Introduction

As one of the major medical resources, acupuncture has been widely used to treat various diseases in China and some other eastern countries for thousands of years. As an alternative and complementary method, acupuncture is gradually accepted in western countries for its undeniable therapeutic effects especially in analgesia [1–5]. Exploring the mechanism of acupuncture has been an active area in alternative and complementary medicine. Since the 1970s, several studies of acupuncture on experimental animals have proven that the integration of central nervous system (CNS) plays an important role in acupuncture efficacy [6]. With the application of multiple neuroimaging techniques such as Positron Emission Tomography (PET) and functional Magnetic Resonance Imaging (fMRI) in acupuncture research, the understanding of the central mechanism of acupuncture has gradually increased [7, 8]. A number of neuroimaging studies indicated that acupuncture could modulate activity in multiple cortical and subcortical brain areas (i.e., somatosensory, brainstem, limbic, and cerebellum) [9]. This included endogenous antinociceptive limbic networks, as well as higher-order cognitive and affective control centers within the prefrontal cortex and medial temporal lobe, and so forth. The cerebral responses elicited by acupuncture stimulation are extensive; therefore it is difficult to determine the underlying mechanism regarding how does each brain region influence one another and how is the relationship among these regions.

In recent years, using the methods and techniques of connectome to explore the functional and structural networks of human brain has become one of the research hotspots in neuroscience [7, 10–12]. The “connectome” holds that the human brain is highly self-organized with regional networks to interconnect and interact. The increasing connectomics studies not only give us a better understanding of the human brain but also make a new approach of revealing the mechanism underlying acupuncture.

In this review, an overview of the basic concept of connectomics will be discussed and the significance of connectomics on central mechanism research of acupuncture will be highlighted. Secondly the commonly used neuroimaging techniques in acupuncture researches will be briefly introduced. Subsequently, the preliminary application of functional connectivity (FC) in acupuncture research will be discussed by reviewing published neuroimaging studies. Finally, the limitation of present research and future direction will be considered.

## 2. The Connectomics

Connectomics is a new research field that has been emerging for studying the structure-function relationship of connectomes among numbers of neuronal elements at all levels, from small microcircuits to cortical columns than to larger areas in the brain [13]. The “connectome” is conceived since the central nervous system (CNS) is considered to realize functional repertoire (i.e., cognition, behavior) via the global and local integration of the brain interconnected networks [14]. These networks are thought to consist of a multitude of functional connected modules (subnetworks) in different hierarchies, which may play an important role in organizing the brain's structural connection. Furthermore, in each structural hierarchy, the module has function of integrating and contextualizing more specialized functions on its submodules. Hence, in order to understand these complex connectomes and reveal the true nature of neuronal interaction in human brain, in July 2009, the Human Connectomics Project (HCP) was launched to set the principle goal of reconstructing the architecture of functional and structural connectivity in human brain by using cutting edge neuroimaging and histological techniques. The connectomics research would not only focus on analyzing and mapping the topology of the structural connectivity, but also on the FC which arises from relatively fixed structures. Specifically, the connectomics draw nodes on the analogy of the interacting brain neural units and the edges on the interconnected links, respectively, trying to observe the heavily connected brain regions. Developed by this mode, the connectomics research has achieved progress in further understanding pathogenesis of some neurological and neuropsychiatric diseases. For example, it has been found that the hippocampus and thalamus exhibit heavily connected, and some rich-club hubs have densely connected nodes in these regions, but after damage, they appears to be vulnerable and reorganized [15, 16]. Another study demonstrated the greatly decreased density of FC among the rich-club hubs in schizophrenia patients [16]. These studies may present the potential relationship between the variation of FC and some diseases.

Recently, by the data analysis such as multivariate Granger Causality Analysis (mGCA) and the whole brain FC analysis of imaging modalities, connectomics research is able to quantitatively characterize the FC among segregated but functionally connected brain regions, which may help to further investigate why the FC is context-sensitive and state-dependent especially in the task-specific state (i.e., attention, memory) although it is constrained by the structural connectivity. Therefore, the connectomics research would have profound influence on neuroscience, which may lead us to a whole understanding of both the human brain activity and its disorders.

Connectomics will significantly influence the central mechanism research of acupuncture in the future, which can be viewed from two perspectives. On the one hand, the regulation of acupuncture is characterized by complexity and holism. Acupuncture can modulate intricate multisystems ranging from peripheral to pivotal. The current neuroimaging data has confirmed that the influence of acupuncture on the CNS is extensive and complex, and the relationship between an acupoint and brain region is not a one-to-one correspondence [17, 18]. Puncturing at a single acupoint evokes multiple brain region responses and puncturing at different acupoints elicits different cerebral activity changes. Therefore, it is unpractical and unconvenient to section each thinly sliced brain tissue to pinpoint the dynamic and diverse regions influenced by acupuncture. And starting with cerebral network and analyzing the correlations of different brain regions would be an important approach in acupuncture research. On the other hand, the brain is the most complex material in nature, and the higher function of the brain is the most complex movement form in nature. Different brain regions are closely interconnected in function and structure by neural connections. By identifying their unique patterns of connectivity, the connectomics is able to analyze the architecture of anatomical and functional connectivity in these brain networks, which may illustrate the functional interconnection among the large-scale brain regions [19]. Hence, the connectomics match the characteristics of acupuncture and the human brain, and the visualized techniques and data processing methods in connectomics will be helpful for exploring the central mechanism of acupuncture by forming cerebral functional-structural networks.

## 3. The Commonly Used Neuroimaging Techniques in Acupuncture Researches

The commonly used neuroimaging techniques in acupuncture researches include MRI, PET/CT, Single-Photon Emission Computed Tomography (SPECT), electroencephalography (EEG), and magnetoencephalography (MEG).

Among these techniques, fMRI, with a high temporal-spatial resolutions, is a predominant technique for observing the FC changes in acupuncture studies [20]. It measures brain activity by detecting associated changes in blood flow. PET is able to measure changes in regional brain functions such as neuronal metabolism or cerebral blood flow by tracing the concentration and distribution of changes of intravenously injected radiolabeled isotope in brain tissue [21]. MEG is based on recording the magnetic field that is generated by active cortical neurons. Unlike PET and fMRI, MEG is able to record neural activity with milliseconds temporal resolution. It can identify the location of this activation with an accuracy level comparable to PET and fMRI [22]. Besides, the Diffusion Tensor Imaging (DTI) is a special MRI technique which can potentially be used to assess the anatomy of white matter (WM) and its connectivity in vivo [23].

## 4. The Influence of Acupuncture on Cerebral Functional Connectivities

**Figure 1:**
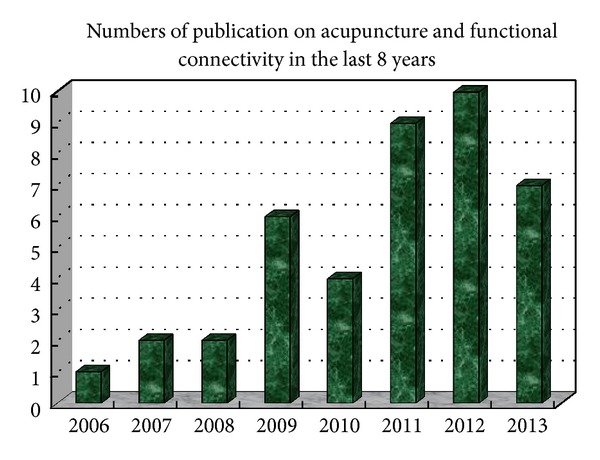


From 2006 to 2013, the number of studies focusing on investigating the cerebral FC changes elicited by acupuncture is increasing year by year. There were totally 41 articles elaborating these studies, with 28 published in English and 13 published in Chinese (shown in Table 1 and Figure 1). Among them, 32 studies were performed on healthy subjects and mainly aimed at investigating the acupoint specificity. The other 9 studies were performed on patients and most of them focused on the therapeutic mechanism of acupuncture. In terms of the intervention, most of these studies used manual acupuncture as their main intervention, and ST36 (Zusanli) is the most frequent acupoint chosen in these studies. These studies have made a progress in exploring the mechanism of acupuncture from a perspective of complex networks and provide references for future application of connectomics in acupuncture study.

### 4.1. Studies on Healthy Subjects

The earliest FC analysis applied in acupuncture studies could be traced back to 2006. Qin et al. [24] were the first to use the seed-based FC analysis to explore the central mechanism of acupuncture. They selected the amygdala as the “seed” and observed the influence of puncturing at ST36 (Zusanli) on FC between amygdala and other brain regions. They ultimately found that the default endogenous analgesia functional network existed in human brain at a low level, and it could be increased by acupuncture modulation.

Subsequently, Hui et al. [25] used fMRI to test the deactivation of the limbic-paralimbic-neocortical network (LPNN) with acupuncture and explore the influence of acupuncture on FC among those regions. They conducted the study by comparing verum acupuncture with tactile stimulation on LI4 (Hegu), ST36 (Zusanli), and LV3 (Taichong) in healthy subjects (HS). They applied seed-based cross-correlation analysis (CCA) to demonstrate the functionally connected brain regions during acupuncture and the acquired results were cross-checked with the model-free probabilistic independent component analysis (pICA). The result indicated that acupuncture may have influence on both functional and structural connectivity of LPNN/the Default Mode Network (DMN). The LPNN/DMN plays a crucial role in keeping functional brain balance and maintaining health. Moreover, the DMN is evidenced to be related to human cognition, affection, and behavior.

Following an increase in studies concentrating on the investigation of FC exerted by the immediate effect of acupuncture, Feng et al. [26] examined a different perspective. The research was focused on detecting whole functional brain networks in the poststimulus period following acupuncture compared to sham acupuncture. The result showed that the limbic-paralimbic regions such as the amygdala, hippocampus, and anterior cingulate gyrus emerged as network hubs following acupuncture but not sham acupuncture. For direct comparisons, increased correlations of acupuncture compared to sham acupuncture were primarily related to the limbic-paralimbic and subcortical regions including the insula, amygdala, anterior cingulate gyrus, and thalamus, whereas decreased correlations were typically related to the sensory and frontal cortices. These results demonstrated that verum and sham acupuncture may exert heterogeneous modulation patterns on the whole functional brain network.

Recently, a study conducted by You and his colleagues combined fMRI and MEG to explore spatiotemporally whether or not band-specific DMN hub configurations would be induced by verum acupuncture, compared with sham control [27]. Spatial independent component analysis was applied to fMRI data, followed by the discrete regional sources seeded into MEG data. Partial correlation analysis was further adopted to estimate the intrinsic FC and network hub configurations. One of the most striking findings of this study is that the posterior cingulate cortex (PCC) was not only validated as a robust DMN hub but served as a hub only within the delta and gamma bands following the verum acupuncture, while PCC was served as a DMN hub in sham control group.

Besides, other studies performed on GB37 (Guangming), KI8 (Jiaoxin), PC6 (Neiguan), PC7 (Daling), CV4 (Guanyuan), CV12 (Zhongwan), and GV20 (Baihui) of HS also identified the influence of acupuncture on cerebral FC [12, 28–32].

### 4.2. Studies on Carpal Tunnel Syndrome Patients

In 2007, Napadow and his coinvestigators explored the influence of acupuncture on patients with Carpal Tunnel Syndrome (CTS) using fMRI and FC analysis [33]. The results indicated that for the baseline CTS patients responding to verum acupuncture, FC was found between the hypothalamus and amygdala—the less the deactivation in the amygdala, the greater the activation in the hypothalamus and vice versa. This study suggested that chronic pain patients responded to acupuncture differently compared to healthy controls, through a coordinated limbic network including the hypothalamus and amygdala.

### 4.3. Studies on Mild Cognitive Impairment Patients

With whole brain FC analysis, Feng et al. found that patients with Mild Cognitive Impairment (MCI) showed abnormal FC in memory-related brain regions including the hippocampus, thalamus, and fusiform gyrus, and acupuncture could significantly influence FC in these abnormal regions [11]. Compared to superficial acupuncture (SA), significantly increased correlations related to the memory-related regions were found with deep acupuncture (DA). They held that deep muscle insertion of acupuncture is necessary to achieve the appreciable clinical effect. With mGCA, the same research team identified that acupuncture at KI3 (Taixi) during different cognitive states and with varying needling depths may induce distinct reorganizations of effective connectivity of brain networks, and DA at KI3 in MCI can induce the strongest and more extensive effective connectivity related to the therapeutic effect of acupuncture in MCI [34].

### 4.4. Studies on Primary Hypertension Patients

With fMRI and within-condition interregional covariance analysis (WICA), Chen et al. found that although short-term acupuncture did not significantly decrease blood pressure, it appeared to decrease body pain and improve vitality. After acupuncture treatment, the hypothalamus-related brain network showed increased FC with the medulla, brainstem, cerebellum, limbic system, thalamus, and frontal lobes [35]. It is believed that acupuncture may regulate the cardiovascular system through a complicated brain network from the cortical level, the hypothalamus, and the brainstem.

### 4.5. Studies on Lumbar Disc Protrusion Patients

With fMRI and seed-based FC analysis, Ye et al. [36] explored the central mechanism of balancing acupuncture technique treating the lumbar disc protrusion. They found that after balancing acupuncture treatment, the patients with lumbar disc protrusion showed increased functional connectivities in multiple regions including the thalamus, brainstem, ventral anterior nucleus, ventral lateral nucleus, medial frontal gyrus, superior frontal gyrus, frontal supraorbital gyrus, inferior frontal gyrus, superior temporal gyrus, middle temporal gyrus, hippocampus, cingulate gyrus, and insula, while HS showed different connectivity changes after treatment.

### 4.6. Studies on Depression Patients

Yi et al. [37] used fMRI and seed-based FC analysis to observe the FC in depressed patients' brain influenced by acupuncture in the resting state. They found that acupuncture could increase the FC between left anterior cingulate and multivarious regions such as the bilateral parietal lobe (left BA40, right BA7), right temporal lobe (BA22), left PCC (BA23), superior frontal gyrus (BA8), left middle frontal gyrus (BA46), and bilateral caudate. These regions were closely related to modulating the emotion of depression.

Furthermore, Li et al. [38] used fMRI and independent component analysis (ICA) to investigate the influence of acupuncture treatment on the FC in DMN in chronic sciatica patients and found sustained effect of acupuncture on modulating the FC in DMN.

Taken together, by using fMRI and FC analysis methods, more and more studies found that acupuncture may have profound influence on extensive regions of the limbic system. Furthermore, acupuncture may have the function of mobilizing the anticorrelated functional networks of the brain, especially deactivating the LPNN/DMN, which may help to explore the central mechanism of acupuncture.

## 5. Limitations and Future Directions 

Although FC has greatly expanded our horizon and enhanced our ability to investigate the central mechanism of acupuncture, it is still at a preliminary stage in connectomics. The limitations in the current studies are as follows: (1) the majority of these studies are performed on HS, while little attention was given to patients. Actually, the therapeutic effect of acupuncture focuses on pathological changes not the physiological condition. Therefore, studies performed on patients are more important for exploring the therapeutic mechanism of acupuncture and (2) among the techniques used in acupuncture research for observing brain FC changes, the fMRI is the most popular. However, it is limited by its indirect nature via BOLD response measurement rather than electrical neuronal activity or substance metabolism. Combining fMRI with other neuroimaging techniques such as MEG, EEG, Diffusion Weighed Imaging (DWI), or PET would be a superior method to improve the results' repeatability in future research and (3) for analysis method, most studies used the whole brain FC analysis and mGCA. Other methods such as “small world” and so forth are also suitable for acupuncture neuroimaging study and (4) for the study design, most studies focused on the immediate effect elicited by acupuncture, while the achievement of acupuncture efficacy usually need a duration of treatment. So investigating the mechanism of the sustained effect or long last effect of acupuncture is more important in the future study and (5) for the quality control of acupuncture neuroimaging, the selection of nonacupoint, the manipulation of manual acupuncture, and the qualification of acupuncturist have effect on result and need for needs for specification and normalization.

## 6. Conclusion

In conclusion, connectomics based on neuroimaging techniques, is one of the forefront of neuroscience. Although the multitarget, multifactorial nature of acupuncture therapy and the current limitations make research in acupuncture central mechanism complex and difficult, we believe that connectomics will provide an important approach for further exploring the central mechanism of acupuncture.

## Figures and Tables

**Table 1:** Studies on the acupuncture and cerebral functional connectivity.

Author	Year	Language	Participants	Case number	Group number	Intervention	Points	Control
Qin et al. [24]	2006	E	HS	14	1	MA	ST36	Baseline versus after acupuncture
Bai et al. [39]	2007	E	HS	8	2	MA	ST36	Acupuncture versus sham acupuncture
Napadow et al. [33]	2007	E	CTS, HS	25 (13 CTS patients)	2	MA	LI-4	Baseline versus after acupuncture CTS patients versus HS
Qin et al. [40]	2008	E	HS	18	2	MA	ST36	Acupuncture versus sham acupuncture
Dhond et al. [41]	2008	E	HS	15	2	MA	left PC6	Acupuncture versus sham acupuncture
Zhang et al. [30]	2009	E	HS	36	3	EA	GB 37, KI 8	EA versus light flash stimulation
Liu et al. [42]	2009	E	HS	56	4	EA	GB37, BL60, KI8, a sham point	Acupuncture versus sham acupuncture
Liu et al. [28]	2009	E	HS	28	2	EA	GB37, KI8	Puncturing at GB37 versus puncturing at K18
Hui et al. [25]	2009	E	HS	48	3	MA	LI4, ST36, LV3	Acupuncture versus superficial tactile stimulation
Liu et al. [43]	2009	C	HS	21	2	MA	ST36	Puncturing at nonacupoints versus puncturing at ST36
Long et al. [44]	2009	C	HS	17	1	MA	ST36	Baseline versus after acupuncture
Zyloney et al. [45]	2010	E	HS	48	4	EA	LI3, LI4	Acupuncture versus sham acupuncture
Qiu et al. [46]	2010	E	HS	38	2	MA	LV3	Female versus male
Ren et al. [32]	2010	E	HS	36	3	MA	PC6, PC7, GB37	Puncturing at PC6 versus puncturing at PC7 versus puncturing at GB37
Hui et al. [47]	2010	E	HS	37	3	MA	LI4, ST36, LV3	Acupuncture versus sham acupuncture
Liu et al. [48]	2011	E	HS	14	2	MA	ST36	Acupuncture versus sham acupuncture
Feng et al. [49]	2011	E	HS	36	3	MA	PC6, PC7, GB37	Puncturing at PC6 versus puncturing at PC7 versus puncturing at GB37
Feng et al. [50]	2011	E	HS	36	3	MA	PC6, PC7, GB37	Puncturing at PC6 versus puncturing at PC7 versus puncturing at GB37
Feng et al. [12]	2011	E	HS	14	2	MA	ST36	Acupuncture versus sham acupuncture
Ye et al. [51]	2011	C	HS	10	1	MA	EX-UE7 (Yaotongdian)	Baseline versus after acupuncture
Ye et al. [51]	2011	C	LIDP, HS	20 (10 HS)	2	MA	EX-UE7 (Yaotongdian)	Baseline versus after acupuncture; LIDP patients versus HS
Ye et al. [52]	2011	C	LIDP	10	1	MA	EX-UE7 (Yaotongdian)	Baseline versus after acupuncture
Li et al. [53]	2011	C	HS	9	1	MA	ST36	Baseline versus after acupuncture
Fang et al. [54]	2011	C	HS	21	1	EA	RN12	Baseline versus after acupuncture
Zhong et al. [55]	2012	E	HS	12	2	MA	GB40, KI3	Baseline versus after acupuncture; puncturing at GB40 versus puncturing at KI3
You et al. [56]	2012	E	HS	28	2	MA	ST36	Acupuncture versus sham acupuncture
Jiang et al. [57]	2012	E	HS	40	2	TEAS		TEAS versus intermittent minimal TEAS
Fang et al. [29]	2012	E	HS	21	2	EA	CV4, CV12	Puncturing at CV4 versus puncturing at CV12
Feng et al. [11]	2012	E	MCI	24	2	MA	KI3	Baseline versus after acupuncture
Li et al. [38]	2012	C	Chronic sciatica, HS	20 (10 HS)	2	EA	GB30, BL40, BL25, BL23, BL57	Chronic sciatica patients versus HS
Zhao et al. [58]	2012	C	HS	20	1	MA	LI4	Baseline versus after acupuncture
Yi et al. [37]	2012	C	Depression, HS	39 (13 HS)	3	MA	LV3	HS versus puncturing at nonacupoints in depressed patients versus puncturing at LV3 in depressed patients
Fang et al. [59]	2012	C	HS	47	3	MA	LV3	Puncturing at LV3 with deqi versus puncturing at LV3 with deqi mixed with sharp pain versus superficial tactile stimulation at LV3
Dai et al. [60]	2012	C	HS	16	1	MA	SP6	Puncturing at nonacupoints versus puncturing at SP6
Zhang et al. [31]	2013	E	HS	12	1	EA	GV20, EX-HN3	5 min versus 15 min after acupuncture
You et al. [27]	2013	E	HS	28	2	MA	ST36	Acupuncture versus sham acupuncture
Jiang et al. [17]	2013	E	HS	18	4	MA, EA, TEAS	ST36	MA versus EA versus TEAS versus sensory stimulation
Dong et al. [61]	2013	E	HS	32	2	NA	NA	Acupuncturist versus nonacupuncturist
Chen et al. [34]	2013	E	MCI	24	2	MA	KI4	Baseline versus after acupuncture
Chen et al. [35]	2013	E	Primary hypertension	30	2	MA	GV20,GV23, EX-HN1 (Sishencong), LI4, ST36, SP6, LR3	Baseline versus after acupuncture
Chen et al. [62]	2013	C	MCI	6	1	MA	DU26	Baseline versus after acupuncture

E: English; C: Chinese; HS: healthy subjects; CTS: carpal tunnel syndrome; MCI: mild cognitive impairment; LIDP: lumbar intervertebral disc protrusion; MA: manual acupuncture; EA: electro-acupuncture; TEAS: transcutanclus electrical acupoint stimulation.

## References

[1] Irnich D, Beyer A (2002). Neurobiological mechanisms of acupuncture analgesia. *Schmerz*.

[2] Wang S-M, Kain ZN, White P (2008). Acupuncture analgesia: I. The scientific basis. *Anesthesia and Analgesia*.

[3] Otti A, Noll-Hussong M (2012). Acupuncture-induced pain relief and the human brain's default mode network—an extended view of central effects of acupuncture analgesia. *Forschende Komplementärmedizin*.

[4] Yu Z, Cao X, Xia Y (2013). Electroacupuncture stimulation at CV12 inhibits gastric motility via TRPV1 receptor. *Evidence-Based Complementary and Alternative Medicine*.

[5] Kerr Grieve J, Flucker S, O’Riordan J (2013). Acupuncture is an effective treatment for pain and other ms symptoms. *Journal of Neurology, Neurosurgery & Psychiatry*.

[6] Han J-S (2003). Acupuncture: neuropeptide release produced by electrical stimulation of different frequencies. *Trends in Neurosciences*.

[7] Liu B, Chen J, Wang J (2012). Altered small-world efficiency of brain functional networks in acupuncture at ST36: a functional MRI study. *PLoS ONE*.

[8] Zeng F, Liu X-G, Tang Y, Liang F-R (2008). Application of PET-CT technique to the research on central mechanism of acupuncture effects. *Zhen Ci Yan Jiu*.

[9] Dhond RP, Kettner N, Napadow V (2007). Neuroimaging acupuncture effects in the human brain. *Journal of Alternative and Complementary Medicine*.

[10] Zhang Y, Glielmi CB, Jiang Y (2012). Simultaneous CBF and BOLD mapping of high frequency acupuncture induced brain activity. *Neuroscience Letters*.

[11] Feng Y, Bai L, Ren Y (2012). FMRI connectivity analysis of acupuncture effects on the whole brain network in mild cognitive impairment patients. *Magnetic Resonance Imaging*.

[12] Feng Y, Bai L, Zhang W (2011). Investigation of acupoint specificity by multivariate granger causality analysis from functional MRI data. *Journal of Magnetic Resonance Imaging*.

[13] Sporns O, Tononi G, Kötter R (2005). The human connectome: a structural description of the human brain. *PLoS Computational Biology*.

[14] Park HJ, Friston K (2013). Structural and functional brain networks: from connections to cognition. *Science*.

[15] van den Heuvel MP, Sporns O (2011). Rich-club organization of the human connectome. *Journal of Neuroscience*.

[16] van den Heuvel MP, Sporns O, Collin G (2013). Abnormal rich club organization and functional brain dynamics in schizophrenia. *JAMA Psychiatry*.

[17] Jiang Y, Wang H, Liu Z (2013). Manipulation of and sustained effects on the human brain induced by different modalities of acupuncture: an fMRI study. *PLoS ONE*.

[18] Bai L, Tian J, Zhong C (2010). Acupuncture modulates temporal neural responses in wide brain networks: evidence from fMRI study. *Molecular Pain*.

[19] Behrens TEJ, Sporns O (2012). Human connectomics. *Current Opinion in Neurobiology*.

[20] Hagmann P, Cammoun L, Gigandet X (2010). MR connectomics: principles and challenges. *Journal of Neuroscience Methods*.

[21] Villringer A (1997). Understanding functional neuroimaging methods based on neurovascular coupling. *Advances in Experimental Medicine and Biology*.

[22] Vrba J, Robinson SE (2001). Signal processing in magnetoencephalography. *Methods*.

[23] Lemaire J-J, Cosnard G, Sakka L (2011). White matter anatomy of the human deep brain revisited with high resolution DTI fibre tracking. *Neurochirurgie*.

[24] Qin W, Tian J, Pan X, Yang L, Zhen Z The correlated network of acupuncture effect: a functional connectivity study.

[25] Hui KKS, Marina O, Claunch JD (2009). Acupuncture mobilizes the brain’s default mode and its anti-correlated network in healthy subjects. *Brain Research*.

[26] Feng Y, Bai L, Ren Y (2011). Investigation of the large-scale functional brain networks modulated by acupuncture. *Magnetic Resonance Imaging*.

[27] You Y, Bai L, Dai R (2013). Altered hub configurations within default mode network following acupuncture at ST36: a multimodal investigation combining fMRI and MEG. *PLoS ONE*.

[28] Liu P, Zhang Y, Zhou G (2009). Partial correlation investigation on the default mode network involved in acupuncture: an fMRI study. *Neuroscience Letters*.

[29] Fang J, Wang X, Liu H (2012). The limbic-prefrontal network modulated by electroacupuncture at CV4 and CV12. *Evidence-Based Complementary and Alternative Medicine*.

[30] Zhang Y, Liang J, Qin W (2009). Comparison of visual cortical activations induced by electro-acupuncture at vision and nonvision-related acupoints. *Neuroscience Letters*.

[31] Zhang G, Qu S, Zheng Y (2013). Key regions of the cerebral network are altered after electroacupuncture at the Baihui (GV20) and Yintang acupuncture points in healthy volunteers: an analysis based on resting fcMRI. *Acupuncture in Medicine*.

[32] Ren Y, Bai L, Feng Y, Tian J, Li K (2010). Investigation of acupoint specificity by functional connectivity analysis based on graph theory. *Neuroscience Letters*.

[33] Napadow V, Kettner N, Liu J (2007). Hypothalamus and amygdala response to acupuncture stimuli in carpal tunnel syndrome. *Pain*.

[34] Chen S, Bai L, Xu M (2013). Multivariate granger causality analysis of acupuncture effects in mild cognitive impairment patients: an FMRI study. *Evidence-Based Complementary and Alternative Medicine*.

[35] Chen H, Dai J, Zhang X (2013). Hypothalamus-related resting brain network underlying short-term acupuncture treatment in primary hypertension. *Evidence-Based Complementary and Alternative Medicine*.

[36] Ye Y, Li B, Chen Z (2011). Resting state-functional magnetic resonance imaging technology applied to a balancing acupuncture treatment for central mechanisms. *Journal of Clinical Rehabilitative Tissue Engineering Research*.

[37] Yi Y, Xu F, Xie P (2012). Acupuncturing Taichong point for regulating the brain function of depression patients:resting-state fMRI study. *China Journal of Traditional Chinese Medicine and Pharmacy*.

[38] Li J, Dong J-C, Yue J-J, Tang W (2012). Effects of acupuncture on default mode network images of chronic sciatica patients in the resting network state. *Chinese Journal of Integrated Traditional and Western Medicine Press*.

[39] Bai L, Tian J, Qin W Exploratory analysis of functional connectivity network in acupuncture study by a graph theory mode.

[40] Qin W, Tian J, Bai L (2008). FMRI connectivity analysis of acupuncture effects on an amygdala-associated brain network. *Molecular Pain*.

[41] Dhond RP, Yeh C, Park K, Kettner N, Napadow V (2008). Acupuncture modulates resting state connectivity in default and sensorimotor brain networks. *Pain*.

[42] Liu P, Qin W, Zhang Y (2009). Combining spatial and temporal information to explore function-guide action of acupuncture using 
fMRI. *Journal of Magnetic Resonance Imaging*.

[43] Liu B, Liu X, Chen J (2009). Study on the effects of acupuncture at acupoint and non-acupoint on functional connectivity of 
different brain regions with functional magnetic resonance imaging. *Chinese Acupuncture and Moxibustion*.

[44] Long Y, Liu B, Liu X (2009). Resting-state functional MRI evaluation of after-effect of acupuncture at Zusanli point. *Chinese Journal of Medical Imaging Technology*.

[45] Zyloney CE, Jensen K, Polich G (2010). Imaging the functional connectivity of the Periaqueductal Gray during genuine and sham 
electroacupuncture treatment. *Molecular Pain*.

[46] Qiu WQ, Claunch J, Kong J (2010). The effects of acupuncture on the brain networks for emotion and cognition: an observation of gender 
differences. *Brain Research*.

[47] Hui KKS, Napadow V, Liu J (2010). Monitoring acupuncture effects on human brain by fMRI. *Journal of Visualized Experiments*.

[48] Liu J, Qin W, Guo Q (2011). Divergent neural processes specific to the acute and sustained phases of verum and SHAM 
acupuncture. *Journal of Magnetic Resonance Imaging*.

[49] Feng Y, Bai L, Zhang W Investigation of acupoint specificity by whole brain functional connectivity analysis from fMRI 
data.

[50] Feng Y, Bai L, Ren Y (2011). Investigation of the large-scale functional brain networks modulated by acupuncture. *Magnetic Resonance Imaging*.

[51] Ye Y, Yang Z, Liu B, Chen Z, Li X (2011). Response of balancing acupuncture by using brain functional magnetic resonance imaging at resting- 
state. *Journal of Jinan University*.

[52] Ye Y, Liu B, Chen Z, Chen J, Liu X, Li X (2011). Brain functional connectivity of balancing technique acupuncture on Backleg pain. *Journal of Liaoning University of TCM*.

[53] Li N, Wang P, Deng B (2011). Influence of acupuncture of Zusanli (ST 36) on connectivity of brain functional network in healthy 
subjects. *Acupuncture Research*.

[54] Fang J-L, Hong Y, Wang X-L (2011). Electroacupuncture at Guanyuan (CV 4) and Zhongwan (CV 12) modulates functional connectivity of the 
brain network in healthy volunteers. *Acupuncture Research*.

[55] Zhong C, Bai L, Dai R (2012). Modulatory effects of acupuncture on resting-state networks: a functional MRI study combining 
independent component analysis and multivariate granger causality analysis. *Journal of Magnetic Resonance Imaging*.

[56] You Y, Bai L, Dai R, Zhong C, Xue T, Wang H (2012). Acupuncture induces divergent alterations of functional connectivity within conventional frequency bands: 
evidence from MEG recordings. *PLoS ONE*.

[57] Jiang Y, Hao Y, Zhang Y (2012). Thirty minute transcutaneous electric acupoint stimulation modulates resting state brain activities: a
perfusion and BOLD fMRI study. *Brain Research*.

[58] Zhao B, Li L, Yang J, Li C, Xu C, Zhu Y (2012). Study on acupuncture effect of Hegu point(LI4)with resting-state fMRI. *Chinese Imaging Journal of Integrated Traditional and Western Medicine*.

[59] Fang J, Hui KK, Liu J, Nixon EE, Zhou K, Wang X (2012). Deqi and Sharp pain during acupuncture at Taichong eliciting the opposite functional brain network 
effects—an fMRI study. *Chinese Imaging Journal of Integrated Traditional and Western Medicine*.

[60] Dai X-J, Min Y-J, Gong H-H (2012). Evaluation of the post-effect of acupuncture at Sanyinjiao (SP 6) under sleep deprivation by resting- 
state amplitude of low-frequency fluctuation: a fMRI study. *Chinese Acupuncture and Moxibustion*.

[61] Dong M, Qin W, Zhao L, Yang X, Yuan K, Zeng F (2013). Expertise modulates local regional homogeneity of spontaneous brain activity in the resting brain: an fMRI 
study using the model of skilled acupuncturists. *Human Brain Mapping*.

[62] Chen S, Xu M, Peng X, Huang J, Yang W, Xu Q (2013). Study of resting-state fMRI in acupuncture at DU26 point in patients with mild cognitive impairment. *Journal of Yunnan University of Traditional Chinese Medicine*.

